# From Magnitudes to Geometry and Back: De Zolt's Postulate

**DOI:** 10.1111/theo.12385

**Published:** 2022-02-13

**Authors:** Eduardo N. Giovannini, Abel Lassalle‐Casanave

**Affiliations:** ^1^ Department of Philosophy University of Vienna Vienna Austria; ^2^ CONICET/Universidad Nacional del Litoral Santa Fe Argentina; ^3^ Department of Philosophy Federal University of Bahia and CNPq Salvador Brazil

**Keywords:** De Zolt's postulate, Euclidean geometry, general magnitudes, Hilbert, logical analysis, plane area, purity of method, whole and parts

## Abstract

A crucial trend of nineteenth‐century mathematics was the search for *pure* foundations of specific mathematical domains by avoiding the obscure concept of magnitude. In this paper, we examine this trend by considering the “fundamental theorem” of the theory of plane area: “If a polygon is decomposed into polygonal parts in any given way, then the union of all but one of these parts is not equivalent to the given polygon.” This proposition, known as De Zolt's postulate, was conceived as a strictly geometrical expression of the general principle of magnitudes “the whole is greater than the part.” On the one hand, we illustrate this striving for purity in the foundations of geometry by analysing David Hilbert's classical proof of De Zolt's postulate. On the other hand, we connect this geometrical problem with the first axiomatizations of the concept of magnitude by the end of the nineteenth century. In particular, we argue that a recent result in the logical analysis of the concept of magnitude casts new light on Hilbert's proof. We also outline an alternative development of a theory of magnitude that includes a proof of De Zolt's postulate in an abstract setting.

A mathematical theory is not to be considered complete until you have made it so clear that you can explain it to the first man whom you meet on the street.

— *David Hilbert* (1990), ‘*Mathematical Problems*’

## 1 INTRODUCTION

The theory of geometrical equivalence investigates criteria for the equality of area of plane polygons by means of their decomposition and composition into polygonal parts, which are congruent in pairs. David Hilbert (1899) formulated the first modern axiomatic version of the theory in his classical *Foundations of Geometry*. As is well known, a fundamental aim of this epochal piece of modern axiomatics was to provide a new independent basis for geometry by delivering a strictly geometrical axiomatization. Accordingly, a central task in the project of developing geometry “out of itself” was to remove any essential (but often implicit) appeal to numerical considerations. This first requirement deviated from the usual “metrical approach” in the theory of plane area, which consisted in measuring the area of polygonal figures by means of (positive) real numbers.

In the same vein, a second methodological requirement — equally important but less explicit — was the exclusion of the general concept of *magnitude* from the axiomatic construction of geometry. This central aspect of nineteenth‐century mathematics is often described as the “the end of the science of magnitude [*Größenlehre*]” (see, e.g., Epple (2003) and Ferreirós (2007)). In this regard, Hilbert's geometrical program in *Foundations* echoed a notable trend of nineteenth‐century mathematics that searched for *pure* foundations of specific mathematical domains by also avoiding the obscure concept of magnitude. In fact, the concept of magnitude was still extremely broad by mid‐nineteenth century; one commonly encountered the definition that magnitude was everything that can be increased or diminished, and can be said to be equal, greater, or lesser. Thus, the term *magnitude* applied not only to geometrical magnitudes but also to different sorts of entities such as numbers, masses, temperatures, time intervals, and the like.

Whereas the requirement of eliminating numbers from the foundations of geometry in Hilbert's early axiomatic work is widely known and often stressed, the problem of developing a “geometry without magnitudes” has largely been ignored in the specialised literature. In the present article, our goal is to begin filling this gap by examining a proposition that might be called the “fundamental theorem” in the theory of plane area, but which is usually known as De Zolt's postulate. A standard formulation of this proposition reads as follows: “If a polygon is decomposed into polygonal parts in any given way, then the union of all but one of these parts is not equivalent to the given polygon” (De Zolt, 1881).

The fundamental role that De Zolt's postulate plays in the development of the theory of plane area can be readily explained. Because this proposition excludes the possibility that a polygon can have less area than itself, it is essential to introduce a (strict) order relation for polygonal areas. From a modern perspective, the role of De Zolt's postulate is to guarantee the existence of a relation of *total* or *linear* order for plane polygons on the basis of the relation of geometrical equivalence. This connection between the central geometrical postulate and a relation of *ordering* for polygonal areas was noted by nineteenth‐century geometers, who took De Zolt's postulate as the mathematically precise formulation, for the case of polygonal areas, of Euclid's famous Common Notion 5 (CN5) in the *Elements*: “The whole is greater than the part.”

From early modern times, this Euclidean principle was conceived as a general principle of magnitudes. Thus, the formulation of De Zolt's postulate was obviously connected with the second aforementioned requirement of purity. At the end of nineteenth century, the common standpoint in elementary geometry was to include De Zolt's proposition as a new geometric axiom, but Hilbert provided a proof of it in *Foundations* satisfying both requirements, that is, avoiding numerical concepts but also general magnitudes. We shall call this approach the *geometrical path* in the theory of plane area.

The alleged “strictly geometrical” formulation of CN5 by means of De Zolt's postulate raised a second problem, which was connected to the modern axiomatic investigations of the concept of magnitude such as Stolz (1885) and Hölder (1901), among others. Roughly, these early axiomatic works conceived magnitude as a combination of an ordered structure and an additive structure. Then, an intriguing issue was whether these abstract characterizations of the concept of magnitude could feature a precise formulation of the central geometrical postulate in strictly *algebraic terms*; moreover, one also could demand that the resulting “algebraic” version of De Zolt's be derived as a *theorem* of an (axiomatic) theory of magnitudes. This problem was addressed some years later, but only very schematically, by Łomnicki (1922). In this paper, we also aim at exploring this less‐travelled “algebraic path” in the theory of area.

Although particularly simple and elegant, the theory of geometrical equivalence hides not only technical challenges but also a rich history of intertwined conceptual problems in the philosophy of (late) nineteenth‐century mathematics, which have been explored in the recent literature on the “purity of method.” The article is written in the spirit of making accessible, if not to the first person whom one meets on the street then at least to a non‐specialist audience, some crossing mathematical paths and philosophical problems in the development of the theory of plane area. Accordingly, in section 2 we introduce the relevant geometrical notions of the theory of equivalence with a minimum of formalism and a maximum of diagrams. Following the same accessibility criterion, in section 3 we distinguish between two different interpretations of the requirement of “purity of method” in connection to the theory of area, and especially to the proof of De Zolt's postulate. In section 4 we reconstruct and analyse Hilbert's geometrical proof of the latter geometrical postulate. Next, in section 5 we explore the connections between the emergence of the modern theory of magnitudes and our geometrical problem, which leads us down the “algebraic path.” Then in section 6 we walk down it by outlining an alternative development of a theory of magnitude that includes a proof of De Zolt's postulate in an abstract setting.

As elementary as this exposition might seem, we believe that it contributes to the current discussion of Hilbert's notion of “purity of method” and to the elucidation of some “algebraic” aspect of his geometrical proof of De Zolt's postulate in *Foundations*. Furthermore, we also offer a more accessible presentation of an alternative “weaker” theory of magnitudes (that we call *compatible magnitudes*), whose salient feature is the inclusion and precise distinction of both CN5 and De Zolt's postulate according to their abstract formulations. This analysis brings then new elements to justify the designation of De Zolt's postulate as the fundamental *theorem* of the theory of plane area.

## 2 AN OVERVIEW OF THE GEOMETRICAL THEORY OF EQUIVALENCE

**FIGURE 1:**
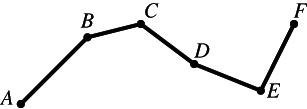
Polygonal segment

**FIGURE 2:**
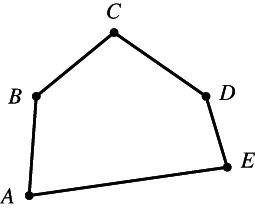
Simple polygon

Although everyone knows what a plane polygon is, let us start with some definitions. A set of line segments such as AB,BC,CD,…,EF is called a *polygonal segment* that *connects* the points A and F. If the points A,B,…,F lie in the same plane and A coincides with F, then the polygonal segment is called a *polygon*. The line segments AB,BC,…,EA are the sides of the polygon, and the points A,B,…,E are its vertices.^1^ Figures 1 and 2 illustrate a polygonal segment and a polygon, respectively.

**FIGURE 3:**
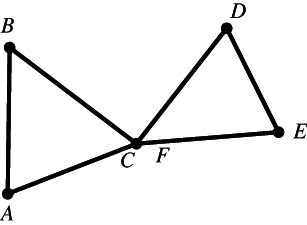
Non‐simple polygon

**FIGURE 4:**
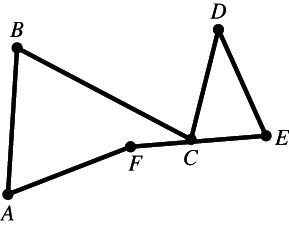
Non‐simple polygon

**FIGURE 5:**
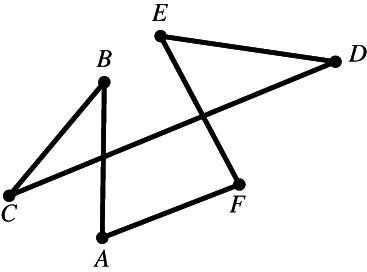
Non‐simple polygon

Figure 2 is a polygon in the most usual sense of the term. Note that by definition, Figures 3, 4, 5 are also polygons.

In what follows, we will deal exclusively with a particular class of polygons known as *simple* polygons, those in which (i) the vertices are all distinct (which is why the polygon in Figure 3 fails to be simple), (ii) no vertex coincides with a side other than the two it is part of (in Figure 4, point C is part of BC and FE), and (iii) no two sides have any interior points in common, as illustrated by the polygon in Figure 5.

Every *simple* polygon divides the plane into an interior and an exterior region. Let us say that a *polygonal region* is the union of a simple polygon and its *interior*. The notion of “area” is concerned with this interior region; intuitively, the area or “content” of a simple polygon is the “size” of the polygonal region determined by it. As we have mentioned, in the context of elementary geometry, there are essentially two main ways of studying polygonal areas: by *measuring* them, that is, by assigning a (positive) real number to every simple polygon corresponding to its area; or by *comparing* them, according to whether one area is larger or smaller than another, or whether the two are equal. Pursuing this second option amounts to developing a theory of geometrical equivalence.

**FIGURE 6:**
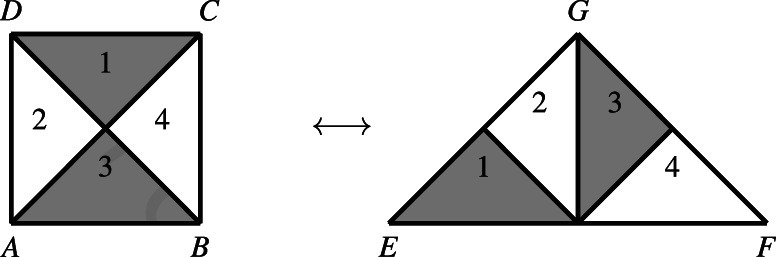
Equidecomposable polygons

**FIGURE 7:**
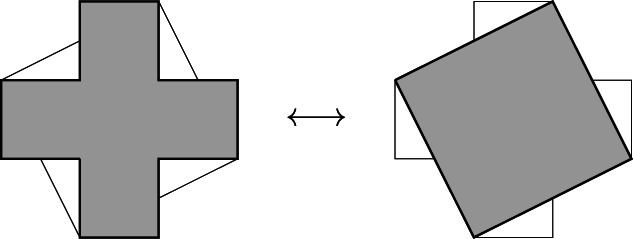
Equicomplementable polygons

Starting with Euclid's treatment of polygonal areas in the *Elements*, we find a purely geometrical technique to establish that two rectilinear plane figures have the same “size” consisting in adding and subtracting other polygons, which are respectively *congruent*. Two polygons are said to be *equidecomposable* if they can be decomposed into the same number of polygonal components which are pairwise respectively congruent, that is, if they are composed of the “same” parts (Figure 6). The relation of equidecomposition is also known as “equivalence by dissection” or “equivalence by congruence.” Moreover, two polygons are called *equicomplementable* if it is possible to “adjoin” to them other equidecomposable polygons, such that the two resulting polygons are also equidecomposable (Figure 7). Thus, the comparison of polygons with respect to their areas is ultimately grounded in the notion of geometrical congruence.

**FIGURE 8:**
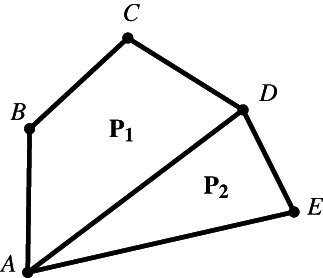
Decomposition of a simple polygon

**FIGURE 9:**
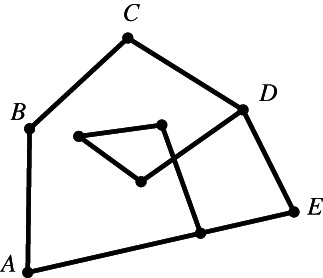
Not a decomposition of a simple polygon

Now, a central notion presupposed in the above definitions of equidecomposability and equicomplementability is that of the *decomposition of a polygon*. Intuitively, we think that a polygon is decomposed into other polygons if it can be expressed as the union of nonoverlapping polygons. For instance, a pentagon can be decomposed into a triangle and a quadrilateral by drawing a diagonal between any two non‐adjacent vertices (Figure 8). But let us consider a more precise definition: a non‐self‐intersecting polygonal segment joining two points of a polygon P and lying entirely in its interior *decomposes* P into two polygons $P_{1}$ and $P_{2}$ with disjoint interiors, each of which is a subset of the interior of P; we also say that $P_{1}$ and $P_{2}$ *compose* P. Figure 9 is not a decomposition of P.

**FIGURE 10:**
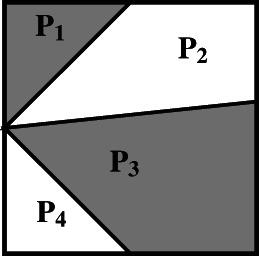
Decomposition into four polygonal parts

In a strict sense, our notion of decomposition stipulates that a polygon can be decomposed by a polygonal segment into *two* other polygons. Naturally, the process of decomposition can be repeated in order to obtain a decomposition of a polygon into n polygonal components. Thus, for example, Figure 10 illustrates a decomposition according to our definition.^2^

Figure 10 seems to illustrate that, in the case of polygonal figures, “the whole is greater than the part” is valid. This general principle is usually known as the famous Common Notion 5 (CN5) in Euclid's *Elements*. But these figures also suggest that all the parts taken together “make up” the whole polygon. And this is explicitly stated in our definition of decomposition of a polygon, where we say that polygons $P_{1}$ and $P_{2}$ *compose* the whole polygon P. Thus, because the operation of addition consists, *intuitively*, in the non‐overlapping union of polygons, our definition also implies that “the whole is equal to the sum of all its parts,” or in symbols:

$$P=P_{1}+P_{2}+\ldots +P_{n},$$

where the equality symbol “=” should be interpreted as the relation of *congruence*.

**FIGURE 11:**
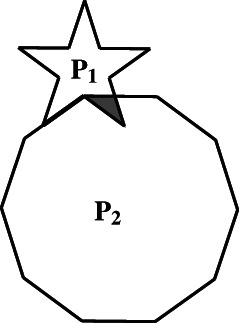
Non‐juxtaposable polygons

Turning to the notion of addition introduced by means of the idea of decomposition of a polygon, we can designate this conception as “local” because the operation is circumscribed or restricted to a particular *given polygon*. Consider the decomposition illustrated in Figure 8, the *geometrical* operation corresponding to the addition of the polygonal parts would consist in the “removal” of the common sides. A main issue is whether this operation can be generalised to any pair of polygons in the sense that it will always be possible to add *any two* given polygons. This comes down to the question of whether any pair of polygons can *always* be juxtaposed. Naturally, the answer is negative: consider a regular starred pentagon and a regular decagon with sides equal or greater to the distance of two consecutive vertices of the pentagon (Figure 11). It is immediately clear that these two polygons cannot share two points along one common side without also having common points in their *interiors*.

**FIGURE 12:**
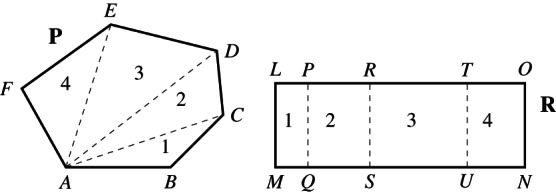
Transformation of a polygon into an equivalent rectangle

A solution to this problem was discovered during Euclid's era and described in his *Elements*. It is a classical theorem studied in high‐school geometry: any polygon is *equivalent* to a parallelogram or rectangle with a given side. Then, any two polygons can always be “transformed” into two equivalent rectangles of equal altitude, which can be trivially juxtaposed and ordered by comparing their bases. More precisely, the procedure to transform any rectilinear figure consists in two simple steps once we know how to transform any triangle into an equivalent rectangle (or parallelogram, in general) with a given side: first, one decomposes the given polygon into triangles (by drawing diagonals from one vertex arbitrarily chosen, for instance); secondly, one transforms these triangles, one by one, into an equivalent rectangle of the same given altitude, placing them side by side (Figure 12).^3^ The fact that the figure thus obtained is indeed a rectangle follows trivially.

Aside from the simplicity of this standard procedure to obtain equivalent polygonal figures, we should stress that the whole method of transformation of areas hinges on a *crucial geometrical fact*. It is clear that once an altitude h is given, rectangles with altitudes h serve to represent the area of any polygon, modulo congruence. Consider now a given polygon P. Because there is no fixed way to decompose a polygon into triangles, there are different ways to apply this method to P. Suppose we have two different decompositions (e.g., triangulations) of polygon P and a given segment h. By applying our method, we obtain two equivalent rectangles $R_{1}$ and $R_{2}$ with the same altitude h. Then, is it absolutely evident that the rectangle $R_{1}$ resulting from one decomposition of P must be *congruent* to the rectangle $R_{2}$ obtained from a different decomposition? Late nineteenth‐century geometers believed that this fundamental fact was not obvious at all, particularly if one considered very *large* but of course finite decompositions and demanded rigorous proof. The requested proof appealed to a very simple reasoning: if the two rectangles were not congruent, then one of the rectangles would be equivalent to a proper part of the other by transitivity of equivalence. Then, this possibility was explicitly ruled out by the formulation of De Zolt's postulate: “if a polygon is decomposed into polygonal parts in any given way, then the union of all but one of these parts is not equivalent to the given polygon.” The postulate excluded the possibility of polygons whose areas are ambiguous according to the method of transformation of areas described above.

From a contemporary perspective, the admission of De Zolt's postulate warrants the introduction of a *total* order relation for polygonal areas based exclusively on the notion of geometrical equivalence. Surely, if a polygonal part could be equivalent to the whole polygon, then polygons will not be comparable with respect to their areas. Nevertheless, a crucial foundational and methodological issue is whether the postulate should be accepted either as a *new axiom* of geometry or proved as the “fundamental” theorem of the theory of equivalence. Interestingly, this pressing question is intimately related to the requirement of the “purity of method” in late nineteenth‐century geometry; we analyse these connections in the next section.

## 3 FROM MAGNITUDES TO GEOMETRY

We have mentioned in the Introduction that Hilbert demanded and provided a rigorous proof of De Zolt's postulate in his *Foundations*. He also asked that such proof satisfied the requirement of the “purity of method.” A classical formulation of purity, due to Hilbert, read as follows: “in the proof of a theorem one *must* use, as far as possible, only the means suggested by the content of the theorem.”^4^ Thus, “purity” was connected with the search of arguments or proofs for mathematical propositions or theorems, for which the means of proofs were considered as appropriate (or inappropriate) in relation to the conditions explicitly stated in such statements. A proof was “pure” if the resources or methods employed were *intrinsic* to, or suggested by the *content* of, the theorem proved or the problem resolved.^5^

But what does “purity of method” *specifically* mean in the context of a proof of De Zolt's postulate? And more generally, what does it mean in relation to the development of the theory of plane area? We can identify at least two interpretations of this methodological requirement, in connection to Hilbert's endeavours to provide a new *independent basis* for geometry, that is, to develop geometry “out of itself.” The first prescribes the explicit avoidance of any fundamental appeal to numerical considerations, especially to the concept of real number, in the axiomatic construction of geometry. In fact, we can reformulate the problem of the possibility of comparing plane polygons in numerical terms. To solve the problem, it is sufficient to show that a numerical (real‐valued) function of area measure exists. Because any two real numbers are always either equal or one is greater (or less) than the other, one only needs to stipulate that:

$$P⋚Q\;\text{if and only if}\;ℱ\left(P\right)⋚ℱ\left(Q\right),$$

where “ℱ” refers to a “measure of area function” that takes (positive) real numbers as values. Polygons are thus totally ordered by means of their numerical measures of area. A pure proof of De Zolt's should then avoid the use of the *standard* notion of measure of area, defined as a real‐valued numerical function.

Moreover, the standard *numerical* definition of area measures depends essentially on the Archimedean axiom or “axiom of measure,” which warrants that real numbers can be used to measure the *length* of every line segment. Informally, this axiom states that given two line segments AB and CD, there is a natural number n such that n copies of AB added together will be greater than CD. Now, the goal of providing a new *pure* foundation for Euclidean geometry did not preclude per se the use of continuity axioms. Nevertheless, one central motivation of Hilbert's geometrical program was to show that a major part of elementary geometry *could* indeed be developed without assuming this group of axioms, particularly the Archimedean axiom. The fact that (full) continuity was not a necessary condition to perform all Euclidean constructions with ruler and compass had been anticipated by Dedekind in his monograph *Was sind und was sollen die Zahlen?* of 1888.^6^

In connection to the theory of plane area, Hilbert proved that the whole theory of geometrical equivalence could be rigorously developed independently of the Archimedean axiom on the basis of the notion of *equicomplementability*. Specifically, he proved that the notions of equidecomposition and equicomplementability were equivalent only in the presence of the latter axiom.^7^ Thus, an adequate proof of De Zolt's postulate should also refrain from using the Archimedean axiom. In other words, the search for a “pure” proof of this geometrical postulate independently of the Archimedean condition was intimately connected to his *ground rule* in *Foundations*, according to which “the principles of the *possibility* of a proof must always be discussed” (Hilbert, 1971, p. 107). Recall that, for Hilbert, the requirement for the purity of methods of proof was “subjective form” of the ground rule. From a historical point of view, to reveal that the latter axiom was not a *necessary* condition to prove De Zolt's postulate was a significant contribution of Hilbert's axiomatization of the theory of plane area.^8^

The second interpretation of “purity” is perhaps less explicit in Hilbert's work but equally important. It is related to a more subtle dimension of his foundational project, namely the exclusion of the general concept of magnitude from his axiomatic reconstruction of Euclidean geometry. One might argue that, in this regard, Hilbert's axiomatization of Euclidean geometry bears many resemblances to Dedekind's program for the foundations of arithmetic in the sense that both projects aimed at excluding the concept of “extensive” or “measurable” magnitude. One of the clearest statements of Dedekind's (1872) “logicist” project in the construction of arithmetic (and analysis) was presented in the following well‐known passage of *Continuity and Irrational Numbers*:

For the way in which the irrational numbers are usually introduced is based directly upon the conception of magnitudes — which itself is nowhere carefully defined — and explains number as the result of measuring such a magnitude by another of the same kind. *Instead of this I demand that arithmetic shall be developed out of itself*. (Dedekind, 1888, pp. 771–770)

The similarity of Hilbert's position with regard to the concept of extensive magnitude can be appreciated in the following remark about the transitive property of the relation of segment congruence:

Often one encounters the conception (possibly, Euclid held also this view) that theorems like the one just proved are pure magnitude relations and, therefore, do not require any kind of particular proof, and should not be introduced as specific geometrical axioms. But this is not correct: this depends entirely on which relations I define as “equal,” “congruent,” and so on. (Hilbert, 1898/1899, p. 320)

In general terms, eliminating the general concept of magnitude from the foundations of geometry involved two main steps: first, general principles of magnitudes should be converted into geometrical propositions by interpreting the relations and operations of magnitudes as specific geometrical relations and operations for every (relevant) kind of geometrical object. The connection with the requirement of “purity” is thus completely evident: assuming that geometrical objects (straight line segments, rectilinear plane figures, solids, etc.) bear all the basic (algebraic) properties of magnitudes was tantamount to accepting without proof that they behave just like “numbers.” In turn, the second step demanded that these geometrical propositions should not be taken as axioms but proved as *theorem* from the axioms of geometry. These two dimensions of the requirement of “purity” characterise what we have called the “geometrical path” in the theory of plane area, that is, the strictly geometrical construction of the theory which avoids not only numerical concepts but also any reference to *general principles* of magnitude. Naturally, both requirements should be met in a “pure” proof of De Zolt's postulate.

It is worth noting that, by the end of the nineteenth century and the beginning of the twentieth century, the first modern axiomatizations of the theory of magnitude laid the groundwork for what is now usually called the “standard theory of magnitudes.” This included the work of Stolz (1885), Hölder (1901), Huntington (1902), and Schatunowsky (1903), among others.^9^ In modern terminology, this theory defines a system of magnitudes as an *ordered commutative (or Abelian) semigroup*. Schematically, in this approach one starts by postulating a relation of equivalence “~” for magnitudes which satisfies the usual properties of reflexivity, symmetry, and transitivity. Then, one introduces a binary operation of addition “+” of magnitudes which satisfies the associative and commutative properties. The first property provides the structure of a *semigroup*; if the second property is also satisfied, the semigroup is called *commutative* or *Abelian*.

A relation of *strict* ordering “_<_” can be introduced with the help of the operation of addition by postulating that this operation also satisfies the comparability property: for any magnitudes a and b, there exists a magnitude c such that either a=b+c or b=a+c or a=b. This condition suggests the following definition of *strict* order: a<b if and only if there exists a c such that b=a+c. The relation of ordering thus defined is a *strict total ordering*.

The domain of magnitudes described by this axiomatic theory, which does not include yet the Archimedean axiom, was often called during this period a system of *absolute magnitudes*. If the latter axiom was also assumed, then one obtained a systems of absolute magnitudes *in the strict sense*.^10^ This “standard theory” of magnitudes constitutes the background of the “algebraic path” in the theory of area, which will be discussed in section 5. But first let us walk down the geometrical path.

## 4 THE GEOMETRICAL PATH

Hilbert's axiomatic construction of the theory of plane area in chapter IV of *Foundations* is a landmark in the development of the modern theory of equivalence. This can be explained due to the fact that many of the fundamental concepts discussed in section 2, such as equidecomposition, equicomplementability, decomposition, and addition of polygons, were introduced in a more precise and rigorous way than ever before. These conceptual refinements are clearly reflected in Hilbert's original proof of De Zolt's postulate.

The central idea of Hilbert's proof was to develop an elementary theory of area measure of polygons in a *purely geometrical fashion*. More precisely, these functions of area measure did not take numerical values as customary; that is, they did not presuppose the possibility of using (positive) real numbers to *measure* the area of polygonal figures by means of the standard formulas. On the contrary, Hilbert defined the measure of area of plane polygons as an *associated* or *characteristic* line segment. The possibility of “measuring” the area of plane polygons using line segments was grounded on a key technical innovation accomplished in chapter III of *Foundations*, namely, the purely geometrical construction of a calculus of line segments. Then, the kernel of the geometrical proof of De Zolt's postulate consisted in appealing to some algebraic properties of this segment calculus to obtain the basic properties of area measure functions and show that equivalent polygons (i.e., equicomplementable) have equal measure of area. Let us analyse Hilbert's proof in a bit more detail.

**FIGURE 13:**
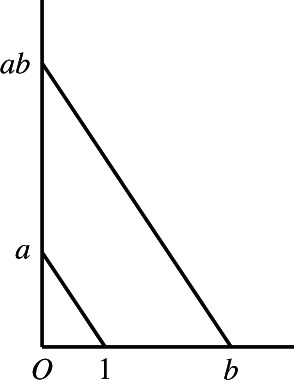
Multiplication of two line segments

The construction of an arithmetic of line segments involved two main steps: first, the purely geometrical definition of the operations of addition and multiplication of line segments; second, the proof that these operations satisfy all the relevant algebraic properties.^11^ More specifically, Hilbert defined the multiplication of two line segments by resorting to the classical construction of the fourth proportional, that is, to the construction exhibited in proposition VI.12 of Euclid's *Elements*. As is well known, this construction had been used for the first time by Descartes to define the product of two line segments as another line segment and demanded fixing a *unit segment* as well as the validity of the parallel axiom (Figure 13). Hilbert also noted that two classical theorems in projective geometry, namely the theorems of Desargues and Pascal, were crucial to obtain important algebraic properties of segment multiplication, such as associativity and conmutativity.^12^ But this led to a notable realisation: the calculus of line segments satisfied all the properties of the (richer) algebraic structure of an *ordered field*.

Let us recall that *ordered fields* are structures of the form $\left\langle F,0,1,+,\cdot ,\leq \right\rangle$ consisting of a set F, an operation of addition +, an operation of multiplication ⋅, and an order relation ≤, such that addition and multiplication are associative and commutative, 0 is the identity of addition, 1 the identity of multiplication, multiplication is distributive over addition, every element has an additive inverse, every element different from 0 has a multiplicative inverse, ≤ is a total order, and the elements greater than or equal to 0 are closed under addition and multiplication. Then, Hilbert's derivation of the algebraic structure of an ordered field from his axioms for the Euclidean plane was not only fundamental for the strictly geometrical introduction of number into geometry but also for the development of the theory of area measure of polygons. Specifically, he defined the measure of area of a polygonal figure as an *element* of this ordered field generated by the segment arithmetic.

It should be noted that Hilbert also used his segment arithmetic to provide an adequate definition of proportionality for line segments and reconstruct the classical theory of similar triangles. More specifically, he defined the proportionality of line segments as the equality of the product of two pairs of line segments, namely: if $a,b,a^{\prime },b^{\prime }$ are any four segments, then the proportion $a:b=a^{\prime }:b^{\prime }$ means nothing else but the segment equation $ab^{\prime }=ba^{\prime }$ (Hilbert, 1971, p. 55).^13^ This definition was central for the geometrical theory of area measure, as we shall see in a moment. Moreover, one central aspect of Hilbert's construction of a segment arithmetic — and of the theory of proportion based on it — was that it does not depend on any continuity assumption, and in particular does not depend on the Archimedean axiom. All these geometrical developments were thus entirely independent of numerical assumptions — and therefore of the concept of real number.

**FIGURE 14:**
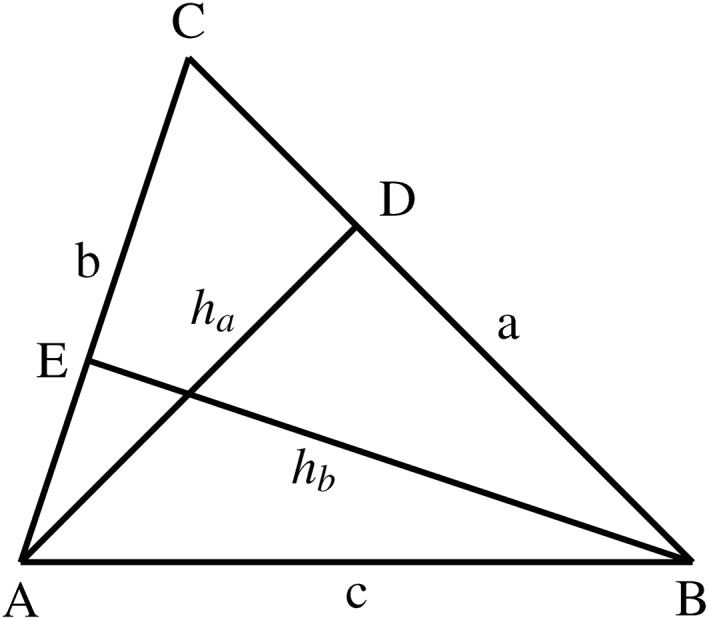
Area measure of a triangle

As usual in the development of a theory of area measure, Hilbert started by first considering the case of triangles. The measure of area of a triangle was defined as a *characteristic segment* obtained through the standard formula, that is, as the semi‐product of the base by the corresponding altitude. Adopting Hilbert's symbolism, $\left[\mathrm{ABC}\right]$ denotes the measure of area of a triangle ABC. The main problem was then to prove that this notion was *well defined* in the sense that the area measure of a triangle is independent of the side chosen as the base and the corresponding heights. Hilbert was able to secure this fundamental property by resorting to his theory of proportion and similar triangles. Two triangles are said to be *similar* if all their corresponding angles are congruent. More precisely, let ABC be a triangle and consider the sides BC=a, CA=b, and the corresponding heights $\mathrm{DA}=h_{a}$ and $\mathrm{BE}=h_{b}$ (Figure 14). From the similarity of the triangles BCE and DCA, it follows that their corresponding sides are proportional, namely: $a:h_{b}=b:h_{a}$. But given the above definition of proportionality, this means that $a.h_{a}=b.h_{b}$, that is, the desired result. It is worth mentioning that this proof presupposed essentially some algebraic properties of segment multiplication, especially the commutative law.

The next step was to assign a *sign* to this notion of area measure based on the definition of an *orientation* for triangles and for polygonal figures in general. The orientation of a triangle (or a polygon) is obtained by considering the order of the corresponding vertices in a clockwise or a counterclockwise direction; the first ordering corresponds to the negative orientation of the figure, the second to the positive.^14^ This amounts to establishing a fundamental property of measure of area functions: the measure of area of any triangle T, positively oriented, is always greater than 0. Additionally, from the fact that the measure of area of a triangle is well defined, Hilbert derived a second fundamental property of functions of measure of area, namely, that congruent triangles have equal measure of areas.^15^

The possibility of decomposing any polygon into triangles suggested the definition of its measure of area: the measure of area of a polygon (positively oriented) is the *sum* of the measure of area of the triangles (positively oriented) in which it is decomposed under a given triangulation. The crucial task in the development of the theory of measure of area of plane polygons was again to prove that this function is well defined, that is, that the area measure is uniquely determined by the polygon or, equivalently, that it is independent of the triangulation used for its calculation. As a matter of fact, this was the part of the proof where the geometrical argument became more complex and involved; Hilbert needed first to derive several auxiliary theorems to achieve the desired result.

We will remain content with pointing out that Hilbert was able to prove, by means of a piece of *purely geometrical reasoning*, that functions of area measure of triangles satisfied the fundamental property of *additivity*: that is, if a triangle is decomposed into a finite number of partial triangles, then the sum of the area measure of the partial triangles (positively oriented) is equal to the area measure the original triangle (also positively oriented). From the additive property of the area measures of triangles, one could prove that the same property was also valid for the measure of area of polygons, and more generally, that every polygon determines uniquely its measure of area independently of the triangulation which employed for its calculation.

Given that congruent polygons have equal area measure, the very definition of a measure of area of polygon allowed one to establish an important geometrical fact: *equivalent polygons (*viz., *equicomplementable) have equal measure of area*. Moreover, the converse, most commonly known as the Wallace–Gerwien–Bolyai theorem, can also be easily obtained from the properties of area measure, especially additivity. Hilbert stated this co‐implication between the relation of geometrical equivalence and measure of area in the following theorem:

Theorem 51 *Two equicomplementable polygons have the same measure of areas and two polygons with the same area are equicomplementable* (Hilbert, 1971, p. 69).

Theorem 51 ensures that if two equicomplementable rectangles have a common side, then their other sides must also coincide. Moreover, this theorem is also often expressed by means of its contrapositive, namely that if two polygons do not have equal measure of area, then they are not equicomplementable. Thus, De Zolt's postulate becomes just a corollary of the latter theorem. Hilbert formulated the fundamental geometrical postulate according to the following version:

Theorem 52 **(De Zolt's postulate)**. *If a rectangle is decomposed by lines into several triangles and one of these triangles is omitted then it is impossible to fill out the rectangle with the remaining triangles* (Hilbert, 1971, p. 69).

Hilbert judged the inference of Theorem 52 to be totally evident, but let us make explicit this final step of his proof of De Zolt's postulate. We need to show that a polygon can never be equicomplementable to a proper polygonal component. Let us consider then a given polygon P, which is decomposed in two or more polygonal parts $P_{1},P_{2},\ldots ,P_{n}$. By the additive property of area measure of polygons, it follows that:

$$\left[P\right]=\left[P_{1}\right]+\left[P_{2}\right]+\ldots +\left[P_{n}\right].$$

However, because the area measure of *each one* of the polygonal parts $P_{1},P_{2},\ldots ,P_{n}$ is greater than 0, the measure of area of the polygon P is *greater than* any of its polygonal parts, such as for example $P_{1}$. Hence, by Theorem 51 P cannot be equicomplementable to $P_{1}$. De Zolt's postulate is a particular case of this result.

With this proof of De Zolt's postulate, Hilbert provides a solid foundation for the theory of equivalence. Specifically, the problem of the *comparability* of plane polygons with respect to the relation of geometrical equivalence (viz., equicomplementability) — which was the central issue concerning this postulate — is solved by the introduction of a notion of *measure of area*. This solution is grounded on the fact that there is a perfect correspondence between the notions of geometrical equivalence and area measure in the case of plane rectilinear figures, as exhibited in Theorem 51 above.

To conclude this section, it is worth stressing again how this geometrical notion of area measure differed from the standard (numerical) one. Hilbert constructed his theory of measure of area independently of the Archimedean axiom, which means that he did not assume the possibility of using (positive) real numbers to *measure* the length of line segments. In a strict sense, his theory consisted in building a *mapping* from the set of plane polygons to the ordered field of the segment arithmetic via the theory of proportion and similar triangles. Furthermore, this appeal to a geometrical construction of a segment arithmetic turned out to be essential for the project of removing the general concept of magnitude from the foundations of geometry, namely: all the “general principles of magnitude” required for the proof of De Zolt's postulate (e.g., additive, commutative, distributive property of multiplication over addition) are regained as basic properties of the *geometrical calculus of segments*. Both demands of “purity” in connection to the proof of De Zolt's postulate were thus met in the geometrical path developed by Hilbert.^16^

## 5 FROM GEOMETRY TO MAGNITUDES

As we have seen, a central motivation for the formulation of De Zolt's postulate was to find a purely geometric version, for the case of polygonal areas, of the *general principle of magnitudes*: “the whole is greater than the part.” The “algebraic path” in the theory of plane area, instead, reverses the question: How can this geometrical postulate be formulated in strictly “algebraic” terms as an axiom or a theorem of an (axiomatic) theory of magnitudes? In fact, this question touches on a crucial conceptual issue for the modern synthetic reconstruction of Euclidean geometry. Even if one of its chief aims was to eradicate the general concept of magnitude from the foundations of geometry, this concept was still a central presupposition of the axiomatic system. To put it concisely: to prove from the axioms of geometry that polygonal areas can be treated as magnitudes, one needs to know first what “magnitudes” are. Paul Bernays (1959) conveys this idea nicely:

The concept of magnitude is, of course, also subjected to axiomatization; however, in this regard the axioms are explicitly separated from the remaining axioms [of geometry] as antecedent. These axioms are of a similar kind as those which are used today for Abelian groups. *But what remained undone*, *because of the methodological standpoint at the time*, *was to determine axiomatically which objects were to be regarded as magnitudes*. (Bernays, 1959, pp. 1–2)

In fact, in section 3 we have seen that a *standard* way to proceed in the development of the concept of magnitude consists in conceiving magnitude as an ordered commutative *semigroup*. This was indeed the common standpoint adopted by the first axiomatizations of the concept of magnitude at the beginning of the twentieth century, which conceived magnitude as a combination of an ordered and an additive structure. Now, it is worth noting that these axiom systems were not formulated with the intention of exploring the logical status of De Zolt's postulate in the modern theory of magnitudes, with the exception of one notable contribution: Łomnicki (1922).^17^ This work laid down a system of axioms conceived explicitly with the chief aim of including an abstract formulation of De Zolt's postulate as a general principle of magnitudes.

Łomnicki claimed that any set of elements could be understood as a magnitude if it is possible to specify three relations, denoted by “=,” “<,” and “>” for these elements such that the following six conditions are satisfied:

1. a=a
2. a=b, then b=a.
3. For every pair of elements, one and only one of the following relations holds:
a<b,a=b,a>b.
4. If a>b, then b<a
5. If a<b and b<c, then a<c.

Łomnicki's system took then a relation of equivalence and a relation of *strict* order as primitives terms. However, his investigation was focused on the formulation of axiom 3, that is, the standard law of trichotomy. More specifically, Łomnicki proved — although only schematically — that in the presence of the other five axioms, the trichotomy axiom can be obtained by means of two *logically independent* propositions, which he called the *completeness of trichotomy* and the *principle of disjunction*:

**Completeness of trichotomy**. *If* a≠b, *then either* a>b *or* a<b.

**Principle of disjunction**. If a=b, then a≮b.^18^

Within this axiomatic framework for an abstract theory of magnitudes, Łomnicki suggested that the latter proposition could be understood as an “abstract version” of De Zolt's postulate. Moreover, he also inquired into the implications of his treatment of the trichotomy law for the development of the geometrical theory of equivalence. His main conclusion was that to guarantee that the set of plane polygons satisfy all the axioms for magnitudes *exclusively by relying on the geometrical theory of equivalence*; that is, without resorting to a notion of measure of area, De Zolt's postulate must be necessarily assumed as an axiom. Łomnicki did not give proof of the notable fact but limited himself to the following informal remark:

Until that axiom [i.e., the principle of disjunction] is satisfied, the geometric relations of equivalence, ‘lesser than,’ and ‘greater than’ of polygons, cannot be regarded as relations of magnitude; and therefore a set of polygons cannot be regarded as a class of magnitudes. Thus, we see what an important role the axiom of disjunction plays, illuminating the dimmest part of the theory of equivalence of polygons (Łomnicki, 1922, p. 25)

Based on the schematic analysis offered by Łomnicki, in a recent paper we have advanced an alternative treatment of the concept of magnitude.^19^ The primary motivation for this approach was to provide a more precise formulation of an *abstract version* of De Zolt's postulate and to derive it as a theorem of this alternative “theory of magnitudes.” In other words, the main goal was to avoid the admission of De Zolt's postulate as an axiom and to prove it as a consequence of the axioms of a “well‐behaved” theory of magnitudes. From this perspective, it is immediately evident that Łomnicki's system is inadequate because it does not include an operation of addition among the primitives. In the next section, we schematically present our alternative analysis of the concept of magnitude and the corresponding treatment of De Zolt's postulate in a more abstract setting. As in other sections of the paper, the emphasis will be put on conceptual issues rather than on technical details.

## 6 THE ALGEBRAIC PATH

We assume as primitives a relation of comparison and an operation of addition of magnitudes. We wish to compare magnitudes such as segments (e.g., straight line segments) with respect to length, and closed figures (e.g., polygons) with respect to area. As basic principles of comparison of magnitudes, we take some familiar properties of ≤. The basic *principles of comparison* ≼ are as follows:

(C.1) Reflexive     for every a: a≼a.

(C.2) Transitive     for all a,b,c: if a≼b and b≼c, then a≼c.

(C.3) Total     for all a,b: a≼b or b≼a.

We introduce *equivalence* ∼ by definition, namely: a∼b iff a≼b&b≼a. Similarly, *strict comparison* is defined as expected: a≺b iff a≼b and a≁b. From totality and the definition of equivalence, we obtain the standard trichotomy law:

Proposition 6.1 (trichotomy). *For magnitudes* a *and* b: a≺b, a∼b *or* a≻b.^20^

**FIGURE 15:**
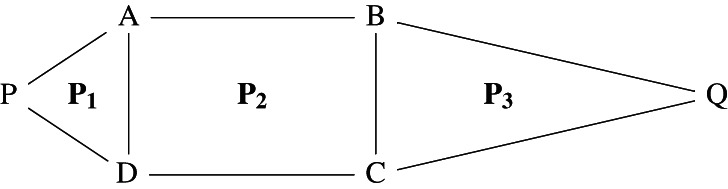
Rectangle ABCD is compatible with triangles PAD and BCQ

The introduction of the operation of addition deserves particular attention. To formulate and prove De Zolt's postulate in this abstract setting, it is convenient to *restrict* the operation of addition to *compatible magnitudes*. On the one hand, this restriction aims to circumvent the problem that any two magnitudes (e.g., plane polygons) cannot always be added. On the other hand, this restriction also attempts to characterise in an abstract way the fact that addition is conceived as a *local* operation. Roughly, the local aspect of addition means that the operation is not performed on any two magnitudes but on magnitudes that are already part of a given magnitude. Compatible magnitudes result from considering in an adequate way a certain decomposition of a previously given magnitude. In a geometrical setting, the idea of compatibility can be illustrated by considering two parts of a given magnitude that have a common element already contained in the latter. For example, two polygonal parts of a polygon are compatible if they have a common side (Figure 15):

We use the symbol “;” to denote the operation of addition. Henceforth we use *compatible* cmp to abbreviate the domain of ;, namely: a cmp b iff, for some magnitude c, we have a;b=c. We assume the following basic *principles of addition* ;.

(A.1) Associativity            for all a,b,c, with a cmp b and b cmp c:

  (a; b) cmp c,          a cmp (b;c),       $\left(a;b\right);c=a;\left(b;c\right)$.

(A.2) Domination      for all a,b, with a cmp b:       a≼a;b≽b.

(A.3) Monotonicities             left and right monotonicities.

(←) Left monotonicity           for a,b,c, with a cmp b and a cmp c:

  if b≼c, then                            a;b≼a;c.

(→) Right monotonicity         for a,b,c, with a cmp c and b cmp c:

  if a≼b, then                         a;c≼b;c.

(A.4) Cancellations             left and right cancellations.

(←) Left cancellation            for a,b,c, with a cmp b and a cmp c:

              if a;b≼a;c, then b≼c.

(→) Right cancellation             for a,b,c, with a cmp c and b cmp c:

              if a;c≼b;c, then a≼b.

A remark about *associative sums* will be relevant for the subsequent discussion.

Remark 6.1 (associative sum). *Given* a,b,c, *with* a cmp b *and* b cmp c, *we have* $\left(a;b\right);c=a;\left(b;c\right)$ *(by associativity **A***. ***1*** *). So*,, *we write simply* a;b;c.

Note also that among the basic principles of addition we have not included the *commutative property*. This is due to the fact that commutativity does not hold in general for our definition of *local* addition of (compatible) magnitudes. Consider, for instance, the local addition of two triangles $P_{1}$, $P_{3}$ and a rectangle $P_{2}$, such that $P_{1}$ shares a side with $P_{2}$ and $P_{2}$ shares a side with $P_{3}$, but $P_{1}$ does not share a side with $P_{3}$ (Figure 15). In this case, the addition $\left(P_{1};P_{2}\right);P_{3}$ is allowed but $\left(P_{2};P_{1}\right);P_{3}$ is not. Thus, it does not result that $\left(P_{1};P_{2}\right)=\left(P_{2};P_{1}\right)$; that is, the commutative property does not hold in general.

We next consider special kinds of magnitudes, that is, *trivial* and *proper* magnitudes, and use this distinction to characterise *strict parts*.

Consider a magnitude m.

1. Call m *left trivial* iff m;b≼b, for some magnitude b with m cmp b.
2. Call m *right trivial* iff a;m≼a, for some magnitude a with b cmp m.

Then, a magnitude m is *proper* iff m is neither right nor left trivial. The distinction between “trivial” and “proper” magnitudes is then established by means of the properties of comparison and addition. The following proposition states that proper addition always causes a strict increase:

Proposition 6.2 (proper concatenation). *Consider magnitudes* m *and* p *such that* p *is proper*.

(←) *If* p *is compatible to* m, *then* m≺p;m.

(→) *If* m *is compatible to* p, *then* m≺m;p.

Proposition 6.2 is an immediate consequence of the trichotomy property. We introduce now *strict parts* of magnitudes, in terms of addition and proper magnitudes. This will be instrumental for a precise formulation of (our abstract version) of Euclid's Common Notion 5. Let us first motivate the notion of strict parts with an example.

**FIGURE 16:**
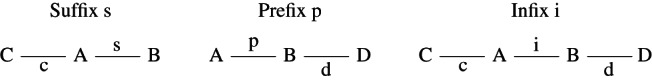
Strict parts of segments

Example 6.1 (strict parts of segments). Figure 16 shows strict parts of segments: a strict suffix s, a strict prefix p and a strict infix i.

Consider magnitudes m and n.

- Call m a *strict suffix of* n iff n=c;m, for some proper c, with c cmp m.
- Call m a *strict prefix of* n iff n=m;d, for some proper d, with m cmp d.
- Call m a *strict infix of* n iff n=c;m;d, for some proper c and d, with c cmp m and m cmp d.

We call m a *strict part of* n iff m is a strict suffix, prefix, or infix of n. Figure 15 shows a rectangle ABCD as a strict part (i.e, a strict infix) of polygon PABQCD. Thus, we have an abstract version of “the whole is greater than the part,” which can be derived as an immediate consequence of proper concatenation (proposition 6.2).

Proposition 6.3 (the whole is greater than the part). *If* m *is a strict part of* n, *then* m≺n.

The next step is to recast the idea of a “decomposition” of a geometrical magnitude in our abstract setting. Recall that in the case of plane polygons, one often establishes the following conditions: a *non‐self‐intersecting* polygonal segment joining two points of a polygon P and lying entirely in its interior decomposes P in two polygons $P_{1}$ and $P_{2}$, each of which is a subset of the interior of P. The generalisation of this notion to the decomposition of a polygon into n polygonal components presents several challenges because it involves, for example, a recursive definition.We analyse the “geometrical” idea of decomposition by means of several notions. We start by considering *magnitude lists* and *truncations*. A magnitude list is a n‐tuple of magnitudes, which we denote as $\underline{a}=\left\langle a_{1},\ldots ,a_{n}\right\rangle$. A truncation of a list is a sublist consisting of all its elements but one: a *head* is obtained by removing the last element; a *tail* is obtained by removing the first element; and a *shell* is obtained by removing another element. We call $\underline{b}$ a *truncation of* $\underline{a}$ iff $\underline{b}$ is a head, a tail, or a shell of $\underline{a}$. Let us illustrate these notions with an example.

Example 6.2 (triangle lists). *Consider the 4‐triangle list* (cf. Figure 15) *as follows*:

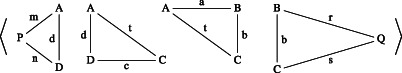

*By removing* BCQ *we obtain its head*

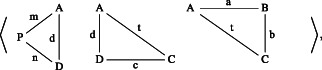

*and by removing* PDA *we get its tail*.

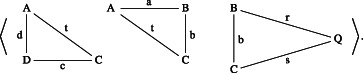

*Moreover*, *it has the following shells*:

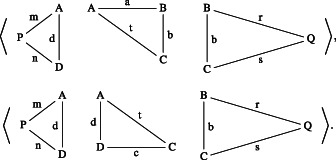

This example suggests that one shall further distinguish a particular kind of magnitude lists that we call *partitions*. More precisely, consider a magnitude list $\underline{a}=\left\langle a_{1},\ldots ,a_{n}\right\rangle$.

- List $\underline{a}=\left\langle a_{1},\ldots ,a_{n}\right\rangle$ is *proper* iff $a_{1},\ldots ,a_{n}$ are all proper.
- List $\underline{a}=\left\langle a_{1},\ldots ,a_{n}\right\rangle$ is *compatible* iff a_1_ cmp a_2_ cmp…cmp a_*n*−1_ cmp a_*n*_.

Then, by a *partition* we mean a proper compatible magnitude list. The next remark is similar to the remark 6.1 about associative sums.

Remark 6.2 (Iterated sums). *For a compatible list* $\left\langle a_{1},a_{2},\ldots ,a_{n-1},a_{n}\right\rangle$, *we have* $\left(\ldots \left(a_{1};a_{2}\right);\ldots ;a_{n-1}\right);a_{n}=\ldots =a_{1};\left(a_{2};\ldots ;\left(a_{n-1};a_{n}\right)\ldots \right)$ *. So*, *we write simply* $a_{1};a_{2};\ldots ;a_{n-1};a_{n}$.

In our example 6.2, notice that the head and tail are compatible, so they are partitions. In turn, the shells are not compatible, so they do not constitute partitions. Furthermore, given a compatible list $\underline{a}=\left\langle a_{1},\ldots ,a_{n}\right\rangle$, its *sum* is the sum of all its elements, namely, the magnitude $\sum \left[\underline{a}\right]≔a_{1};\ldots ;a_{n}$. For n=1, we take $\sum \left[\left\langle a_{1}\right\rangle \right]≔a_{1}$.

We introduce now our “abstract” notion of *decomposition* in terms of partitions. This will be instrumental in formulating and deriving abstract versions of De Zolt's postulate. In particular, we distinguish between local and global decompositions. Given a magnitude m, consider a partition $\underline{a}=\left\langle a_{1},\ldots ,a_{n}\right\rangle$ with sum $\sum \left[\underline{a}\right]$. We call partition $\underline{a}$:

(~) a *global decomposition* of m iff $\sum \left[\underline{a}\right]∼m$,(=) a *local decomposition* of m iff $\sum \left[\underline{a}\right]=m$. In a geometrical setting, one can illustrate this distinction by considering the case of plane polygons and interpreting the symbols “=” and “∼” as congruence and equivalence (e.g., equidecomposition), respectively. Thus, given a partition $\underline{d}$ of a polygon P, if the sum of all its elements yields a polygon Q *congruent* to P, then d is a local decomposition. In contrast, if the sum of all the elements of $\underline{d}$ yields a polygon R *equivalent* to P, then d is a global decomposition.

We next consider truncations of decompositions, which lead us to the following key result:

Proposition 6.4 (partition truncation). *Given a partition* $\underline{p}$, *consider a truncation* $\underline{q}$ *of* $\underline{p}$ *. Then*, *either* $\underline{q}$ *is not compatible or* $\sum \left[\underline{q}\right]≺\sum \left[\underline{p}\right]$.

In fact, given a partition $\underline{p}=\left\langle p_{1},\ldots ,p_{n}\right\rangle$, consider for instance its head $\underline{q}=\left\langle p_{1},\ldots ,p_{n-1}\right\rangle$. It is clear that $\sum \left[\underline{q}\right]$ is a *proper strict prefix* of $\sum \left[\underline{p}\right]$, that is, the magnitude $\sum \left[\underline{q}\right]$ consisting of the sum of all but one of the elements of the partition $\underline{p}$ is a *strict part* of the magnitude $\sum \left[\underline{p}\right]$. But because “the whole is greater than the part” (proposition 6.3), we have that $\sum \left[\underline{q}\right]≺\sum \left[\underline{p}\right]$. The same reasoning can be applied to other possible kinds of truncations (viz., tails and shells), provided they constitute partitions of the magnitude $\underline{p}$; that is, all their elements are proper and compatible (see Example 6.2).

We thus obtain our abstract versions De Zolt's postulate as two main theorems about global and local decompositions:

**De Zolt's postulate** (global decompositions) *Consider a global decomposition* $\underline{p}$ *of* m *. Then*, *for each truncation* $\underline{q}$ *of* $\underline{p}$: $\underline{q}$ *is not a global decomposition of* m.

**De Zolt's postulate** (local decompositions) *Consider a local decomposition* $\underline{p}$ *of* m *. Then*, *for each truncation* $\underline{q}$ *of* $\underline{p}$: $\underline{q}$ *is not a local decomposition of* m.

The “global version” of De Zolt's follows immediately from the above property about partition truncation (proposition 6.4) and our definition of *strict order* ≺: If $\sum \left[\underline{p}\right]∼m$, then (as $\sum \left[\underline{q}\right]≺\sum \left[\underline{p}\right]$), we have $\sum \left[\underline{q}\right]\;∼\;m$. In turn, the “local version” rests on the fact that given a magnitude m, every local decomposition of m is a global decomposition of m.

The similarities between this “abstract proof” of De Zolt's postulate and Hilbert's geometrical proof are then manifest. Recall that the latter proof relied on the fact that two polygons with equal area measures are always equivalent (viz., equicomplementable) and on two fundamental properties of area measures, such as they are always positive and satisfy additivity. This particular treatment of De Zolt's postulate, only sketched here, contributes to clarifying certain key steps in Hilbert's proof of the central proposition in the theory of equivalence of polygons.

Finally, we should point out that our formal version of Common Notion 5 can be derived from the above theorems about global and local decompositions, thereby establishing tight connections between the abstract versions of De Zolt's postulate and “the whole is greater than the part.” Furthermore, both De Zolt's postulate and Common Notion 5 are obtained without assuming the commutative property of addition, which constitutes a difference with respect to the usual analysis of the concept of magnitude. It is worth mentioning that neither have we assumed the Archimedean axiom; this was a leading motivation in Hilbert's proof of the former proposition.

## 7 CONCLUSION

As Hilbert (1899) pointed out in the final section of *Foundations*, the demand for the purity of method was a central methodological requirement in nineteenth‐century mathematics. On the one hand, purity primarily constituted an *ideal of proof*. During this period, it was common to grant a privileged place to proofs that employed only resources which were intrinsic or inherent to the theorem proved or the problem solved, that is, proofs that relied on methods suggested by the “content” of the proposition under consideration. On the other hand, purity was also tightly bound with the search for independent or *autonomous* foundations for different mathematical theories or even whole mathematical fields. In this regard, the central concern was to avoid and eliminate any (often implicit) reference to “foreign” or “exogenous” concepts when laying down the foundations of a mathematical domain.

Our discussion of De Zolt's postulate or the “fundamental theorem” of the theory of plane area, as we have called it here, has contributed to display some overlooked aspects of these intertwined dimensions of the requirement of purity of method. Along with the exclusion of arithmetical or numerical concepts, Hilbert's “pure” (axiomatic) construction of Euclidean geometry essentially demanded the elimination of the general concept of magnitude. By the end of the nineteenth century, the precise formulation of the basic principles that characterised this concept has not been entirely achieved. Nevertheless, the general principle of magnitudes “the whole is greater than the part” was *in some form* included in every axiomatization of the concept of magnitude. The formulation and subsequent proof of De Zolt's postulate as the strictly geometric version of Euclid's CN5 responded to this demand of eliminating the general concept of magnitude from the foundations of geometry. The quest for a purely geometric proof of this postulate was not only a matter of the Dedekindean dictum that “in science nothing capable of proof ought to be believed without proof” (Dedekind, 1888, p. 790); it was also a critical part of a foundational program in geometry similar to the project of eliminating the concept of extensive magnitude from the foundations of arithmetic and analysis.

We have also identified and briefly analysed a different and less explored trend in the modern axiomatic investigations of the concept of magnitude. This trend called for a formulation of De Zolt's postulate in strictly algebraic terms and for the construction of a theory of magnitude that included this abstract version of the postulate as a theorem. This “algebraic path” inspired several clarifications and conceptual refinements. In our schematic exposition, we distinguished between “local” and “global” addition of magnitudes and restricted the operation of addition to “compatible” magnitudes. We explained the idea of the decomposition of a geometrical magnitude in abstract algebraic terms; this helped to cast light upon the conceptual relations between abstract versions of CN5 and De Zolt's postulate.

Finally, our reconstruction of the abstract proof of De Zolt's postulate clarifies certain “algebraic” aspects of Hilbert's proof, which are relevant for discussing the problem of the purity of proofs in mathematical practice. The latter proof relies essentially on the geometrical introduction of area measure functions, although this notion is not explicitly invoked in the “statement of the theorem.” From Hilbert's methodological standpoint, the recourse to this metrical concept of area was not problematic because it was independent of the Archimedean axiom and, *a fortiori*, of the concept of real number. Nevertheless, our distinction between proper and trivial magnitudes contributes to elucidating the detour through a notion of area measure in his proof of the “fundamental theorem” of the theory of geometrical equivalence.

## Footnotes

^1^
We adopt here Hilbert's definition of polygon based on the idea of a *closed* polygonal segment or broken line (Cf. Hilbert, 1971, pp. 8–9). Note that this definition does not make explicit that no two intermediate segments must be collinear, that is, that no three consecutive intermediate vertices must lie on the same line. For a discussion of alternative definitions of polygons, see Grünbaum (2012).

^2^
A more precise definition then demands a *recursive* definition of decomposition into several polygons.

^3^
This procedure was developed by Euclid in the propositions I.42–45 of the *Elements* and is usually known as the method of “applications of areas” (cf. Heath 1956). For an excellent study of Euclid's theory of area, see De Risi (2020).

^4^
Hallett & Majer (2004, p. 217. Our emphasis).

^5^
The requirement of purity of method, especially in the context of modern axiomatic geometry, has recently been the focus of important studies. See, for instance, Hallett (2008) and Arana and Mancosu (2012). For more general discussions of this methodological requirement in mathematics, see Arana and Detlefsen (2011).

^6^
Cf. Dedekind (1888, p. 793). Note that up to the seventh edition of *Foundations* of 1930, Hilbert employed a version of the Archimedean axiom that explicitly avoided the reference to the concept of natural number. Furthermore, a similar concern can be identified in his adoption of a *full* continuity axiom, namely his famous (geometrical) axiom of completeness. On several occasions, Hilbert stressed that this axiom was strictly geometrical because it avoided analytic concepts such as the notions of a limit and convergence. Another salient feature of Hilbert's axiom of completeness is that it does not imply the Archimedean axiom as a logical consequence. For an illuminating discussion of purity and the adoption of (full) continuity axioms in elementary geometry, see Baldwin (2018b).

^7^
Hilbert achieved this original result by constructing a model of a non‐Archimedean geometry where central theorems about the equidecomposition of triangles and parallelograms with equal bases and altitude did not obtain. Following Baldwin (2018b), we could claim that this result plays a significant role in specifying the *axiomatic context* that is necessary for assessing the “content” of De Zolt's postulate.

^8^
It is worth mentioning that many important geometers, such as F. Schur, O. Stolz, and W. Killing, provided detailed proofs of De Zolt's postulate during the 1890s. However, all these proofs assumed explicitly (or more often implicitly) the Archimedean condition. In his lecture course on Euclidean geometry of 1898/1899, Hilbert emphasised on several occasions that these geometers failed to meet the fundamental point, namely that the postulate is probable without Archimedean axiom (Cf. Hilbert, 1898/1899). For a historical discussion of these developments and a detailed analysis of Hilbert's proof, see Giovannini (2021).

^9^
For a detailed historical study of the emergence of the modern theory of magnitudes, see Ehrlich (2006). For a more philosophical discussion of the “standard” theory of magnitudes, see Stein (1990) and Hale (2000).

^10^
Cf. Stolz (1885, p.70). Towards the end of the nineteenth century, there were also important attempts to provide precise (axiomatic) characterizations of the concept of an abstract domain of magnitudes (or quantities), which could be used to construct the real numbers as relations (ratios) between magnitudes. Naturally, this aim required to impose additional structure on the systems of absolute magnitudes in the strict sense (i.e., totally ordered Archimedean Abelian groups). Briefly, these systems of absolute magnitudes were also required to be *densely ordered* and *complete*. See, for example, Hölder (1901), and Frege (1903). For a detailed examination of Frege's constructions of the real numbers and his concept of a domain of magnitudes, see Hale (2000) and Panza and Sereni (2019).

^11^
Hilbert's construction of an arithmetic of line segment is often stressed as one of the most innovative technical contributions of *Foundations*. For detailed studies, see Baldwin (2018a, 2018b) and Giovannini (2016).

^12^
To be more precise, Hilbert proved that while the theorem of Desargues is essential to obtain the associative law of multiplication, the theorem of Pascal guarantees the commutative property of the same operation.

^13^
It might be worth recalling how Hilbert's treatment of segment multiplication differs from Descartes' original ideas. As is well known, Descartes *derived* his definition of segment multiplication from the proposition VI.2 of the *Elements* on the proportionality of similar triangles; consequently, he assumed not only the classical theory of proportion of Book V in its entirety but also the validity of the Archimedean axiom. In contrast, Hilbert regained *directly* the notion of proportionality by starting from his definition of segment multiplication. This was fundamental to circumvent the appeal to the latter axiom in the theory of proportion and similarity.

^14^
Note that in Hilbert's plane geometry the notions of clockwise and counterclockwise can only by defined in a *relative sense*; that is, only *ordered* non‐collinear points can be said to be the same or different with respect to their orientation. There is no way within his geometrical theory to identify the orientation of a triple of points as clockwise or counterclockwise in an absolute sense. We would like to thank an anonymous referee for this observation.

^15^
It is worth mentioning that Hilbert's introduction of *oriented* areas was also relevant for metageometrical reasons. In the appendix II of *Foundations of Geometry*, Hilbert proved that if the usual triangle congruence axiom (III.5) is replaced by a *weaker* version, which limits its application only to triangles with the same *orientation*, several basic propositions of the Euclidean theory of area fail. Most notably, the proposition I.39 of the *Elements* as well as De Zolt's postulate do not obtain if the weaker version of the triangle congruence axiom is assumed. To prove this fact, Hilbert constructed a model of a “non‐Pythagorean geometry,” that is, a geometry in which the Pythagorean theorem does not hold either. For details, see Hilbert (1902/1903).

^16^
Regarding Hilbert's proof of De Zolt's postulate, Hartshorne (2000) has observed that “this proof is analytic in that it makes uses of the field of segment arithmetic, and similar triangles. We do not know any purely geometric proof, for example of (I.39), that triangles on the same base with equal content [i.e., equicomplementable] have the same altitude” (p. 2010). A similar remark has been made by Baldwin (2018b). As for the status of the segment arithmetic, it is clear that Hilbert conceived it as a purely geometrical result. He based this claim on the fact that the elements of the associated fields were strictly geometrical entities and that the definitions of addition and multiplication were “purely geometrical concept formations” [*geometrische Begriffsbildung*] (Hilbert, 1898/1899, p. 374), which did not rely on any kind of number or proportion among numbers. Thus, he did not consider that the use of the segment arithmetic introduced impure elements in his proof of De Zolt's postulate. Nevertheless, the presumed impossibility of proving De Zolt's with elementary means raises interesting and complex issues for the general distinction between synthetic and analytic proofs in geometry, which needs to be addressed in a different paper.

^17^
Łomnicki's paper consists of a detailed summary of conference at the Polish Philosophical Society in Lviv on 21 January 1922. An English translation of this talk by Andrew McFarland can be found in Giovannini et al. (2019).

^18^
The reformulation of trichotomy in terms of a conditional was a suggestion made my J. Lukasiewicz. The name *principle of disjunction* clearly refers to the definition of the conditional by means of the disjunction connective. See Łomnicki (1922, p. 24).

^19^
The next section aims to present a more informal or intuitive exposition of the abstract treatment of magnitudes developed in Giovannini et al. (2019). For historical details of Łomnicki's work, and particularly for the context of this conference, see McFarland et al. (2014, pp. 51–55). Łomnicki's talk was attended by Ajdukiewicz, Bad, Twardowski, and Banach; the latter will start an essential collaboration with Tarski the following year. In particular, in a famous collaborative paper of 1924, Banach and Tarski pursued the notion of geometrical equivalence in much greater detail, particularly in three dimensions. This paper contained a remarkable and “paradoxical” result: two balls with any radii whatever are equivalent — for example, the earth and a marble. This line of inquiry leads to a notable result in three dimensions, namely: there is not a perfect correspondence between the (metrical) concept of volume and the notion of geometrical equivalence (viz., equidecomposition) because there are polyhedra (viz., tetrahedra) with equal volume but which are not equivalent.

^20^
The proofs of all the propositions that follow are given in Giovannini et al. (2019).
